# Central nervous system antiretroviral efficacy in HIV infection: a qualitative and quantitative review and implications for future research

**DOI:** 10.1186/1471-2377-11-148

**Published:** 2011-11-22

**Authors:** Lucette A Cysique, Edward K Waters, Bruce J Brew

**Affiliations:** 1 Departments of Neurology and HIV Medicine, St. Vincent's Hospital, Sydney, Australia; 2 Brain Sciences, St. Vincent's Hospital Clinical School, faculty of Medicine, University of New South Wales, Sydney, Australia; 3 St Vincent's Centre for Applied Medical Research, Sydney Australia; 4 Biostatistics & Databases Program, Kirby Institute (formerly National Centre in HIV Epidemiology and Clinical Research), University of New South Wales, Sydney, Australia

## Abstract

**Background:**

There is conflicting information as to whether antiretroviral drugs with better central nervous system (CNS) penetration (neuroHAART) assist in improving neurocognitive function and suppressing cerebrospinal fluid (CSF) HIV RNA. The current review aims to better synthesise existing literature by using an innovative two-phase review approach (qualitative and quantitative) to overcome methodological differences between studies.

**Methods:**

Sixteen studies, all observational, were identified using a standard citation search. They fulfilled the following inclusion criteria: conducted in the HAART era; sample size > 10; treatment effect involved more than one antiretroviral and none had a retrospective design. The qualitative phase of review of these studies consisted of (i) a blind assessment rating studies on features such as sample size, statistical methods and definitions of neuroHAART, and (ii) a non-blind assessment of the sensitivity of the neuropsychological methods to HIV-associated neurocognitive disorder (HAND). During quantitative evaluation we assessed the statistical power of studies, which achieved a high rating in the qualitative analysis. The objective of the power analysis was to determine the studies ability to assess their proposed research aims.

**Results:**

After studies with at least three limitations were excluded in the qualitative phase, six studies remained. All six found a positive effect of neuroHAART on neurocognitive function or CSF HIV suppression. Of these six studies, only two had statistical power of at least 80%.

**Conclusions:**

Studies assessed as using more rigorous methods found that neuroHAART was effective in improving neurocognitive function and decreasing CSF viral load, but only two of those studies were adequately statistically powered. Because all of these studies were observational, they represent a less compelling evidence base than randomised control trials for assessing treatment effect. Therefore, large randomised trials are needed to determine the robustness of any neuroHAART effect. However, such trials must be longitudinal, include the full spectrum of HAND, ideally carefully control for co-morbidities, and be based on optimal neuropsychology methods.

## Background

The possibility that some antiretroviral drugs with more efficient Central Nervous System (CNS) penetration as part of Highly Active Antiretroviral Therapy (HAART) may be associated with better neurocognitive (NC) functioning and more efficient cerebrospinal fluid (CSF) HIV RNA suppression than other ARVs has important clinical and therapeutic implications [1]. In this paper, we will refer to more efficient CNS HAART as neuroHAART.

First, if true, it indeed means that a non-negligible number of individuals with HIV-associated neurocognitive disorders (HAND) are not receiving optimal treatment. Without proactive assessment of HAND, the individuals with asymptomatic neurocognitive impairment (ANI) and Mild Neurocognitive Impairment (MND) [2], which now represent the greatest proportion of HAND (as opposed to HIV-associated dementia; HAD in the pre-HAART era) are likely not to be considered for a specific therapeutic strategy. A French study [3] brings support to this potential sub-optimal treatment scenario. This study found that in the pre-HAART era, individuals with HAD were preferentially treated with antiretrovirals with greater CNS penetration. However, this was still sub-optimal as it used pre-HAART drugs. They then found that in the HAART era, the treatment strategy as assessed retrospectively, did not favour neuroHAART for individuals with HAD, while ANI and MND were not considered at all.

Second, and perhaps most importantly, there are currently no HAART guidelines for HAND [4]. While this topic is hotly debated [5], still an informative review of the literature has been missing.

Lastly, there is the prospect that some individuals at risk for HAND may benefit from preventative treatment. This question is currently being studied in an international large-scale trial: http://insight.ccbr.umn.edu/start/

The possible superior efficacy of neuroHAART, however, remains highly controversial in the HIV research community because studies have provided conflicting results. More importantly, the definitions of what constitutes neuroHAART vary (see [1] for review of neuroHAART definitions). The underlying premise that the brain is an HIV sanctuary site less amenable to effective systemic treatment is itself at the centre of a scientific debate [6]. However, evidence for the central nervous system (CNS) being a virological sanctuary site can be drawn from clinical [7], CSF [8-10] and human neuropathological studies [11,12].

The aim of this review is to better synthesise the results of existing studies conducted to address the issue of the potential superior efficacy of neuroHAART on brain functions and CSF HIVRNA suppression. This review is based on an innovative staged review strategy which was designed to overcome study design and neuroHAART definition variations. First, each study was reviewed using a blind quality scoring for the presence or absence of major design, methodological and analysis features (adapted from [13]) combined with a non-blind assessment of appropriate use of demographic and longitudinal (practice effect) corrections on neuropsychological data, as well as test sensitivity to HAND. Studies with at least three methodological limitations were excluded (incidentally retaining studies within the upper confidence interval limits of the quality scoring distribution). Remaining studies were then evaluated for statistical power, that is their ability to detect a significant difference based on their stated aims. The implications of the findings are discussed for future research.

## Methods

### Study selection and data extraction

Individual studies were retrieved *via *a search of Pubmed with the following keywords here presented in alphabetical order: antiretroviral, CSF, cognitive functions, CNS, HAART, HIV RNA, HIV-associated dementia, HIV-associated neurocognitive disorders, HIV/AIDS, index, neuropsychological functions, penetrance, penetration.

The following combinations were used: 1. HIV-associated dementia and antiretroviral, CNS, penetrance, penetration, index, HAART, CSF, HIV RNA. 2. HIV-associated neurocognitive disorders and antiretroviral, CNS, penetrance, penetration, index, HAART, CSF, HIV RNA. 3. Neuropsychological functions, HIV/AIDS, and antiretroviral, CNS, penetrance, penetration, index, HAART, CSF, HIV RNA. 4. Cognitive functions, and antiretroviral, CNS, penetrance, penetration, index, HAART, CSF, HIV RNA.

The following criteria were then used to select studies:

1. Reports had to be conducted in the HAART era (that is after 1996).

2. Group comparisons had to have subject numbers of 10 or more; lower numbers provide unstable effect sizes.

3. Reports had to investigate the effect of more than one single drug on an existing HAART regimen because the current review was focused on multiple ARV agents' effect.

4. Studies had to not be based on retrospective data analyses. Some of those studies not only included a sub-optimal definition of HAND, but also were prone to systemic biases in the baseline clinical status of individuals starting a more or less efficient neuroHAART regimen [14].

Using these criteria 16 studies were identified and they are detailed in Table 1. Seven were excluded and their references are included in additional file 1.

**Table 1:** Review of studies that have assessed the effect of CNS penetrating ARTs on NP performance and/or on CSF HIV RNA

*Author & date*	What	Samples	HIV Disease	Design	Findings	Quality scoring > 80% *	< 80% Quality scoring Main factors	POWER > 80%
**Antinori et al., **2002 [25]	CSF 39% detectable viral load at baseline	75 advanced HIV+ individuals 37% naive 29 advanced HIV+ individuals	39% AIDS Median current CD4: 131	Cross-sectional Longitudinal Initiating cART or new cART/retest mean: 11 weeks	*Indinavir associated with greater HIV RNA suppression in the CSF* *Greater CSF HIV RNA suppression with 3 or more CNS penetrant ARTs*	**No**	Clinical groups heterogeneous with multiple types of CNS HIV-related disorders IVDU risk factor in 40%	-

**Chang et al., 2003 **[26]	NP tests CSF 97% detectable viral load MRS	33 HIV+ individuals all ART naïve 19 with HAD	Mean current CD4: 182	Longitudinal 3 months follow-up NP tests MRS	*Better NP performance in individuals on 2 CNS penetrant drugs on 2 NP tests* No correlation between number of CNS penetrant ARTs and reduction in MRS abnormalities.	**Yes**	-	**No**

**Cysique et al., 2004 **[27]	NP tests	97 advanced HIV+ individuals on long-term CART (average 5 years) 100% AIDS	Mean Nadir CD4: 73 Mean current CD4: 369	Cross sectional	*Better performance in Learning and memory when on a CART regimen with = > 3 neuroactive agents in NP impaired (N = 26)*	**Yes**	-	**No**

**Cysique et al, 2006 **[28]	NP tests	81 advanced HIV+ individuals on long-term CART (average 5 years) 100% AIDS	Mean Nadir CD4: 73 Mean current CD4: 385	Longitudinal Yearly for an average of 27 months	*Improvement on Psychomotor speed* *on a CART regimen with = > 3 neuroactive agents*	**No**	Inclusion/exclusion criteria not readily available; NeuroHAART definition not readily available	-

***Author & date***	**What**	**Samples**	**HIV** **Disease**	**Design**	**Findings**	Quality scoring > 80% *	**< 80% Quality scoring** **Main factors**	**POWER > 80%**

**Cysique et al. 2009 **[29]	NP tests CSF 85% detectable at baseline	37 HIV+ individuals with mild to moderate HAND Initiated on CART 38% ART naïve	Means Nadir CD4 = 106 Baseline CD4 = 195 AIDS 77%	Longitudinal Every 12 weeks for 48 weeks	*Overall improvement in cognitive functions with higher CPE*	**Yes**	-	**No**

**De Luca et al., 2002 **[30]	CSF Median log _10 _CSF HIV RNA: 2.9	95 HIV+ individuals On cART 50 HIV+ individuals On cART	Median current CD4: 110 Median current CD4: 59	Cross-sectional Longitudinal Follow-up median of 7 weeks	Higher number of CNS penetrant ARTs correlated with lower CSF HIV RNA *(trend only)*. *Greater longitudinal decrease in CSF HIV RNA associated higher number of CNS penetrant*	**No**	Clinical groups heterogeneous with multiple types of CNS HIV-related disorders IVDU risk factor in 30-40%	-

**Eggers et al., 2003 **[31]	CSF 80% detectable at baseline	40 HIV+ individuals 10 with HIVE 8 with HAD	Median current CD4: 60 29% CDC stage C	Longitudinal LP prior and after cART initiation Unclear time-frame	No correlation between the number of CNS penetrant drugs and slope of CSF viral decay.	**No**	Definition of HAND using brief screens Clinical groups heterogeneous	-

**Marra et al., 2003 **[32]	NP tests CSF 75% detectable at baseline	25 HIV+ individuals HAND baseline rate?	Mean current CD4: 259	Longitudinal Testing before CART initiation at 4 & 8 weeks after Comparison of regimen containing AZT & IDV to other regimen	*Improved on 4 NP tests associated with VL suppression in the CSF in ART naïve *(but not 8 weeks) No significant change in CSF viral load.	**No**	Small test battery Unclear inclusion/exclusion criteria Unclear baseline level of NP-impairment No adequate normative data No practice effect correction	-

***Author & date***	**What**	**Samples**	**HIV** **Disease**	**Design**	**Findings**	Quality scoring > 80% *	**< 80% Quality scoring** **Main factors**	

**Marra et al., 2009 **[33]	NP tests CSF Median log _10 _CSF HIV RNA at baseline: 3.3	101 HIV+ individuals initiating or changing cART	Median CD4: 111	Longitudinal Follow-up at 24 and 52 weeks ACTG 736	*Odds of suppression of CSF HIV RNA were higher when CPE rank was = > 2 (N = 79)* **Impaired HIV+ individuals on a cART with a CPE = > 2 had worse NP performance over time (N = 26) on NP 4 tests**, but not 8 NP tests.	**No**	Unclear inclusion/exclusion criteria Short NP testing battery Lack of education and racial correction in NP tests relevant to the study population	-

**Letendre et al., 2004 **[34]	CSF Mean log _10 _CSF HIV RNA at baseline: 4.1	31 HIV+ with mild to moderate HAND	81% AIDS Means nadir Cd4: 30 Current CD4: 111	Longitudinal Testing before & 15 months after CART initiation	*Greater CSF HIV RNA reduction with higher number of CNS penetrant ARTs*	**No**	Unclear study time points No control for practice effect Correlational analyses only No practice effect correction	-

**Letendre et al., 2008 **[35]	CPE CSF 17% detectable at baseline	467 HIV+ individuals on cART 389 Undetectable and 78 Detectable	77% AIDS Medians nadir CD4: 116 current CD4: 406	Cross-sectional Validation of the CPE index	*CPE < 2 associated with an 88%* *increase in the odds of detectable CSF viral load* *CPE ranks were associated with detectable CSF viral loads with and without treatment and disease adjustments*	**Yes**	-	**No**

**Patel et al., 2009 **[36]	Survival time	2398 HIV+ children 77 incident HIVE [incidence rate 5.1 cases per 1000 person-years.	CD4% ≤ 15%: 19%	Longitudinal Median 6.4 years AACT219/219C	*High CNS-penetrating regimens associated with a survival benefit (74% reduction in the risk of death, 95% CI 39-89%) after HIVE diagnosis compared with low CNS-penetrating regimens*	**No**	Clinical groups heterogeneity Clinical diagnoses as outcome No NP assessment	-

***Author & date***	**What**	**Samples**	**HIV** **Disease**	**Design**	**Findings**	Quality scoring > 80% *	**< 80% Quality scoring** **Main factors**	

**Sacktor et al., 2001 **[37]	NP tests	18 in single in CSF penetrant group 55 in multiple CSF penetrant group With psychomotor slowing 6-7% HAD	11%-31% AIDS Mean current CD4: 339-255	Longitudinal Six annual study visit cART initiation	No difference in NP improvement between 2 groups.	**No**	Unclear inclusion/exclusion criteria NeuroHAART definition not readily available Short NP battery	-

**Sevigny et al., 2004 **[38]	Incident HAD	203 advanced non-demented HIV+ individuals 73% on cART	Median current CD4: 127	Longitudinal Median follow-up of 21 months 36% with incident HAD	Regimens containing = > CNS penetrant ARTs was not associated with time to HAD	**No**	Clinical groups heterogeneity Ad hoc analyses of time to HAD Time to HAD not a validated measure of NP change	-

**Smurzynski et al., 2011 **[39]	NP tests	2636 HIV+ individuals at least 6 weeks on cART	Median current CD4: 243 Nadir CD4: 182	Longitudinal Median follow-up of 4.7 years CPE rank score & ARTs in cART Neuroscreen: 3 NP tests	*When cART was composed of more than 3 ARTs there was a positive association between CPE and better NP performance in unadjusted and adjusted models*.	**Yes**		**Yes**

**Tozzi et al., 2009 **[40]	NP tests	Patients with (n = 93) or at risk for (n = 92) HIV-associated neurocognitive disorders	37% stage CDC C Mean current CD4: 292 Nadir CD4: 181	Cross-sectional Longitudinal NP testing before and after cART initiation (20 months mean interval) Comparison of 2 "neuropenetration" scores (CPE vs. numbers)	*Higher CPE correlated with better NP performance at baseline and follow-up*, but not using the number of CNS penetrant drugs	**Yes**	-	**Yes**

NB: *italicized font*: beneficial effect of NeuroHAART on NP performance and or CSF HIV RNA reduction

Regular font: neutral effect of the NeuroHAART,

**Bold front**: negative effect of NeuroHAART.

ARTs: Antiretrovirals

LP: lumbar puncture

HIVE: HIV encephalopathy

MRS: Magnetic Resonance Spectroscopy

MND: Minor motor Deficits

MSK: Memorial Sloan Kettering

NP: neuropsychological

A score less than or equal to 80% meant that a study presented at least three or more methodological limitations.

#### Qualitative analysis

### Blinded and non-blinded review for quality scoring

Because the quality (as assessed by statistical power, sample size, reliability of assessment of HAND, definitions of CNS penetration efficiency (CPE) score, and neuropsychological methods) of studies varied substantially, we computed a score to rank each study on these factors. This scoring form was adapted from [13]. The statistical methods and design used in each study were reviewed blindly by E.K.W who was provided with a printed copy of the studies (after removal of authorship identifiers and without the title, the abstract and the discussion) and a scoring form (additional file 2). In brief, we used 15 quality criteria that fell into 5 categories (Blinded: design, outcomes, subjects, controls, and un-blinded outcomes). A not-applicable option was provided for criteria that might not apply for all studies (e.g., it made no sense to examine whether there were demographic differences between cases and controls in observational studies with no control arm). Importantly, a non-blinded review of the presence or absence of neuropsychological cross-sectional norms, longitudinal norms (correction for practice effect) and validity and sensitivity of tests used to assess NC performance in HAND (including size of the neuropsychological battery) was then performed by a neuropsychologist (L.A.C.). This review was non-blinded because it sometimes required the exploration of previous papers by the same research team. Therefore a choice was made that all papers would be treated equally with a non-blind assessment.

As mentioned above, in some instances, quality criteria were "not applicable" to some included studies. To account for these cases and still rank the study on a similar scale, a total percentage score was developed. This total percentage score was computed based on the aforementioned criteria and studies were ranked (see Table 1). A score less than or equal to 80% meant that a study presented at least three or more significant methodological limitations. This continuous score was developed to be more transparent than assessing quality categories such as "poor", "medium" or "high" quality (additional file 3).

Whilst our qualitative analysis necessarily involved subjectivity, when presented with studies of poor or variable quality some subjective assessment is needed to ensure that *like is compared with like*; a well designed RCT should ideally not be grouped with a retrospective observational study in such an analysis. Whilst Finney argued in a seminal paper on meta-analysis that an assessment can be purely qualitative when studies are heterogeneous in nature or provide low quality evidence, [15] our scoring method enabled us to transparently identify qualitatively similar studies and analyse them further to provide some quantitative conclusions.

#### Quantitative analysis

### Power computations

**Figure 1:**
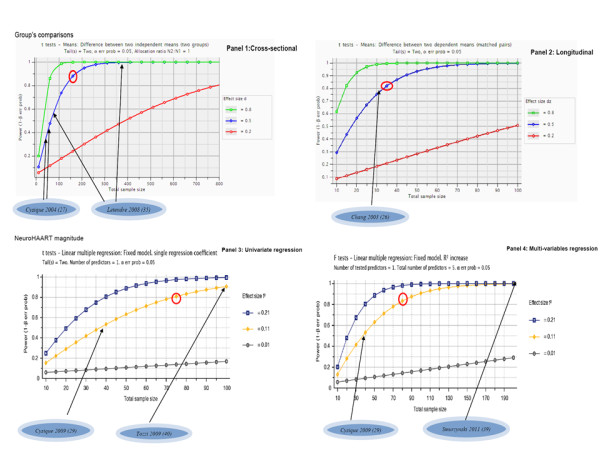
**Power projections**. The figure provides power projections for four different study designs in order to assess NeuroHAART effect on neurocognitive or CSF HIVRNA suppression (i.e., panel 1 illustrates a cross-sectional design; panel 2 a longitudinal design; panel 3 an univariate regression model design, and panel 4 a multi-variable regression model design). The first part of the power projection is dedicated to sample's comparisons and the second part to the testing of the magnitude of the neuroHAART effect on neurocognitive functions or CSF HIV RNA. For each panel, power projections were computed to detect a small, medium and large effect size. In each panel, the six studies found to be of "appropriate quality" were rated against the power's projection for their relevant design and arrows indicates the N enrolled for each study. In addition a red circle in each power panel indicates the criterion against which studies were defined as having "appropriate versus non-appropriate power" and this was selected for the medium effect size.

The objective of the power analysis was to determine the study's ability to assess its proposed research aims. Accordingly, we developed the following strategy: the power computations were conducted [16] using GPower version 3.1 [17] for the studies with a quality scoring greater than 80%. The power projections were made for conventionally small, medium and large effect sizes (namely, 0.20; 0.50 and 0.80) [18]; and were made separately for cross-sectional and longitudinal designs and univariate and multivariate designs (see Figure 1). The studies were then checked against an "acceptable" criterion: power of 80%; two-tailed with a *p*-value less than 0.05. Using this criterion for a medium effect size (*d *= 0.50), we selected which studies in the review were conducted with acceptable power, though it should be acknowledged that the definition of acceptable power is inherently subjective [19].

## Results

The quality scores from the 16 studies were normally distributed with a mean of 76.6% and a standard deviation of 12.8% (additional file 3). Of the 16 studies analysed, six had less than three methodological limitations, and none obtained a quality of 100% which would have reflected an optimal design for addressing their research aims (see Table 1). Four of the six studies retained were longitudinal; all included individuals who had been well characterised neuropsychologically and clinically to determine the full HAND spectrum, while using appropriate normative data when required. The four retained longitudinal studies included statistical methods taking into account the potential practice effect associated with repeated neuropsychological testing. All these studies found a positive effect of NeuroHAART on NC yielding small to large effect sizes (additional file 4). One cross-sectional study and one longitudinal study also found that CSF HIVRNA was more effectively suppressed as a function of a higher CPE.

### Studies' quality scoring highlights (see also Table 1)

#### Heterogeneity of study samples

A number of studies included individuals with various HIV-associated neurological conditions. Others did not carefully report inclusion or exclusion criteria. A few studies did not provide enough information to assess the baseline rate of neuropsychological impairment in their cohort. Lastly, convenience comparisons were sometimes used between a sample on and off NeuroHAART while these samples differed on a number of important clinical characteristics.

#### Insensitive assessment tools in the case of neurocognitive studies

Among the studies investigating NC functions, five (out of 11) included either a small number of tests or clinical scales rather than standard neuropsychological assessment. Others used non-cognitive endpoints such as time to HAND and survival time, which do not directly address potential change in NC functions, but only represent surrogates that are less sensitive to neuropsychological change.

#### Lack of norms and practice effect correction

One study did not use adequate normative data correction for demographic factors potentially misclassifying impairment rate at baseline. Among the neuropsychological longitudinal studies, 50% did not correct for practice.

#### Assessment time points

One major point in the heterogeneity of the published study designs was the variation in test retest intervals. Among studies with higher quality scoring, only one used a short test retest at 3 months. The four others used at least a 12 month retest interval.

### Studies quantitative analysis highlights (see also Table 1)

#### Power

Using our criterion for "appropriate power" we found that two of the six studies with higher quality scoring were conducted with appropriate power (medium effect size that is d = .50; 80%, 2-tailed with *p *= .05). Figure 1 also illustrates which criterion was used for the four different study designs).

### Other major issues in all studies

#### Non-reported p-values or data to compute effect sizes

The majority of studies reported p-values or data allowing effect sizes' conversion when appropriate. Two studies did not provide exact p-values or data for their non-significant findings (see legend for Table 1).

#### Various definitions of neuroHAART

Studies used various definitions of neuroHAART (see Table 1) sometimes including less than the conventional definition for HAART without good rationale (i.e., less than three ARVs in some comparisons). Seven studies used a simple continuous aggregate of ARVs with good penetration. Moreover, five of 16 studies (excluding the CNS Penetration Effectiveness (CPE) validation study) have used what has been termed the CPE score. This is an empirically derived score that is a summed aggregate of the individual scores for each ARV in a HAART regimen.

## Discussion

Our search criteria identified 16 studies that addressed the issue of neuroHAART efficacy.

Qualitative analysis of these studies for the presence of less than three major methodological limitations (which incidentally corresponded to selecting studies within the 5% upper bound of the Student *t *distribution) showed that 37.5% (6 out of the 16) met the criterion for "higher quality". The main methodological limitations, which are relatively common to the field of clinical research in NeuroAIDS, were clinical heterogeneity of the sample studied or compared, unclear inclusion/exclusion criteria, insensitivity/brevity of the neuropsychological battery in 45% of studies, and lack of correction for practice effect (this was found in 50% of longitudinal studies). Other less common limitations were lack of normative correction for baseline demographic factors, and the definition of neuroHAART not fully described to allow direct replication.

Among the studies that scored above the quality scoring cut-off, it should be noted that none achieved a 100% score. This reflects the practical and scientific constraints of clinical research studies. One study did not provide clear enough inclusion/exclusion criteria; one did not provide a clear neuroHAART definition for direct replication; one used a medium-size neuropsychological battery and one a very brief neuropsychological battery; one included comparison groups with a lack of clinical homogeneity, and one did not include correction for practice effect. It should be noted here that the blind review allowed applying the same strictness to the studies' evaluation for all studies.

Moreover, our quantitative analysis on the remaining six studies which met an adequate quality scoring showed that two met the criterion for 80% *a priori *power. The advantage of our staged strategy was that only studies with a higher quality, hence greater comparability could be assessed in the quantitative phase.

Altogether, this review clearly demonstrates that methodological limitations and in most studies, lack of power render the literature difficult to readily interpret without both the qualitative and quantitative approach outlined in this paper. In favour of a positive NeuroHAART effect are the six studies with higher quality scoring, however most are underpowered and none were randomised. Nonetheless, all six controlled for factors that may have been affected by non-randomization using multivariate analyses and still yielded results in favour of a positive neuroHAART effect on NC functions or CSF HIVRNA suppression.

While these results are not definitive they may assist the clinician in decision making as no negative effect of NeuroHAART was predominant. It seems reasonable, therefore, to consider neuroHAART whenever possible in a patient with HAND, notwithstanding issues of adherence and resistance to particular ARVs. However, the most definitive answer to the issue of the potential superior efficacy of neuroHAART remains randomised controlled clinical trials conducted in different regions of the world to provide cumulative evidence.

Limitations of the current qualitative and quantitative review are that it was based only on published studies and did not account for any publication bias towards only reporting positive effects. However, because the existence of any neuroHAART effect is an area of great uncertainty in NeuroHIV and HIV/AIDS research, we contend that a negative finding is of substantial interest and as likely to lead to a publication as a positive finding. Finally it could be argued that our qualitative phase should have selected a stricter level of quality. However, as discussed above, a stricter level of quality would have excluded almost all studies form the quantitative analysis. Moreover, despite methodological limitations, observational studies are an important phase of clinical research, providing preliminary evidence regarding treatment efficacy.

From our analysis of existing studies there are several key aspects that should be considered in the design of a potential future trial.

1. *A priori *power analyses should be conducted for the ability to detect at least a medium effect size. This implies that a future trial should include at least 100 subjects in each arm (see also Figure 1; multivariate graph). This also takes into account the full HAND spectrum including at least mild neurocognitive disorder (MND) [2] and that adequate power is needed to detect a relatively small effect (the treatment effect) in individuals who are not demented, but do have neurocognitive difficulties.

2. Adequate outcome measures should be selected. Even if a brief assessment is selected, it should be targeted to include tests of psychomotor speed, working memory as well as learning and memory to have a wider range of potential benefit from neuroHAART [20]. Related to this issue, and because of the high complexity of the neuropsychological data in this type of study, the inclusion of a senior neuropsychologist in the research team is essential. Moreover, the use of neuro/psychology graduates for a high quality and standard administration of neuropsychological instruments including computerised battery is highly recommended. Because the *NC measure represents the main outcome *of those studies, special care in the data collection and data management should be a basic requirement. This will reduce measurement error and systematic biases that are likely with a poorly trained staff or staff who do not have the basic clinical skills to examine patients with cognitive impairment.

3. Adequate sampling: the study should carefully consider what types of patients are to be included with clear inclusion and exclusion criteria. Principally, the current HAART status and HAART duration should be considered, (See [21] for extensive consideration on this issue). Lastly, if the study includes a test of HIV RNA change, a baseline level of detection may be set as an inclusion criterion to improve the homogeneity in each arm on this aspect. Other aspects that may be considered to improve arm homogeneity are HIV duration, nadir CD4, and previous HAART history.

4. Adequate study time points: the study should be *longitudinal *and select an *early follow-up *to detect HIV RNA changes in the CSF and plasma as well as NC change (between 4 and 7 weeks after treatment initiation) *and, a longer follow-up *ideally at about 48 weeks, to detect long-term NC change. The risk of a shorter term trial is to produce negative or neutral findings when actually a positive effect is at play [21].

5. Adequate analytical strategies: in a randomised trial, which by design minimize systematic biases between treatment arms on the outcome measures, extra consideration in the use of neuropsychological normative data to determine baseline impairment rate is needed. In some instances adaptive randomization may offer a flexible solution (see [22] for further discussion on this issue).

5. We would recommend the use of the CPE score pending improved definitions and over older definitions. This would allow planning preliminary analyses with the version of a current CPE at the time of enrolment in future clinical trials. Uniform use of the CPE score would make direct comparisons of the regimens used in different studies easier and enable a larger evidence base to define the most effective neuroHAART regimens to be compiled. In the future definitions, the role of a potentially impaired BBB should also be considered [23]. Also, the adverse effect of some ARVs on the cardio-vascular system would need to be taken into account in the new version of a CPE score as they have been associated with NC impairment in the HIV population [24].

## Conclusions

Studies assessed as using more rigorous methods found that neuroHAART was effective in improving neurocognitive function and decreasing CSF viral load, but only two of those studies were adequately statistically powered. Because all of these studies were observational, they represent a less compelling evidence base than randomised control trials for assessing treatment effect. Therefore, large randomised trials are needed to determine the robustness of neuroHAART effect. However, such trials must be longitudinal, include the full spectrum of HAND, ideally carefully control for co-morbidities and be based on optimal neuropsychology methods.

## List of abbreviations

CNS: Central nervous system; HAND: HIV-associated neurocognitive disorder; HAART: Highly Active Antiretroviral Therapy; CSF: cerebrospinal fluid; CPE: CNS penetrance efficiency; ANI: asymptomatic neurocognitive impairment; MND: Mild Neurocognitive Impairment; NC: neurocognitive; RCT: randomized control trial; BBB: blood brain barrier.

## Competing interests

Dr. Lucette Cysique has received honoraria from ViiV Healthcare and Abbott

Prof. Brew has received honoraria from ViiV Healthcare, Abbott, and Merck.

## Authors' contributions

LAC has designed the study, conducted the studies' search, conducted the non-blind review, conducted the statistical analyses under the supervision of EKW, written the first draft of the paper, finalize the manuscript. EKW has conducted the blind review, overseen the statistical analyses, and participate to the writing of the manuscript. BJB has participated in the study design and the writing of the manuscript. All authors read and approved the final manuscript.

## Pre-publication history

The pre-publication history for this paper can be accessed here:

http://www.biomedcentral.com/1471-2377/11/148/prepub

## Supplementary Material

Additional file 1 **Excluded studies per our criteria of exclusion**. detailed references of the excluded studies. Click here for file

Additional file 2 **Quality scoring table and instructions for NeuroHAART studies**. This table and instructions are an exact reproduction of those designed for the blind review of studies. Click here for file

Additional file 3 **Quality scores distribution in the 16 observational NeuroHAART studies**. Figures and details of the quality scores obtained by each study. Click here for file

Additional file 4 **Details of the effect sizes' computations**. Figure providing the effects sizes in the 6 studies with a quality score > 80%. Click here for file

## References

[B1] Wright E Neurocognitive impairment and neuroCART Curr Opin HIV AIDS 2011 2011 5 10.1097/COH.0b013e3283477c46 21546833

[B2] Antinori A Arendt G Becker JT Brew BJ Byrd DA Cherner M Clifford DB Cinque P Epstein LG Goodkin K Updated research nosology for HIV-associated neurocognitive disorders Neurology 2007 69 18 1789 1799 10.1212/01.WNL.0000287431.88658.8b 17914061 PMC4472366

[B3] Lanoy E Guiguet M Bentata M Rouveix E Dhiver C Poizot-Martin I Costagliola D Gasnault J Survival after neuroAIDS: association with antiretroviral CNS Penetration-Effectiveness score Neurology 2011 76 7 644 651 10.1212/WNL.0b013e31820c3089 21248274

[B4] Antiretroviral therapy http://www.who.int/hiv/topics/treatment/en/

[B5] ASHM Proceedings Report - Antiretroviral Guidelines Consensus Discussion (2010) ASHM: 2010; Sydney, Australia 2010 ASHM http://www.ashm.org.au/default2.asp?active_page_id=25216789083

[B6] Dahl V Josefsson L Palmer S HIV reservoirs, latency, and reactivation: Prospects for eradication Antiviral Res 2009 4 4 10.1016/j.antiviral.2009.09.016 19808057

[B7] Robertson KR Smurzynski M Parsons TD Wu K Bosch RJ Wu J McArthur JC Collier AC Evans SR Ellis RJ The prevalence and incidence of neurocognitive impairment in the HAART era AIDS 2007 21 14 1915 1921 10.1097/QAD.0b013e32828e4e27 17721099

[B8] Cunningham P Smith D Satchell C Cooper DA Brew B Evidence for independant development of resistance to HIV-1 reverse transcriptase inhibitors in the cerebrospinal fluid AIDS 2000 14 1949 1954 10.1097/00002030-200009080-00010 10997399

[B9] Staprans S Marlowe N Glidden D Novakovic-Agopian T Grant RM Heyes M Aweeka F Deeks S Price RW Time course of cerebrospinal fluid responses to antiretroviral therapy: evidence for variable compartmentalization of infection AIDS 1999 13 1051 1061 10.1097/00002030-199906180-00008 10397535

[B10] Strain MC Letendre S Pillai SK Russell T Ignacio CC Gunthard HF Good B Smith DM Wolinsky SM Furtado M Genetic composition of human immunodeficiency virus type 1 in cerebrospinal fluid and blood without treatment and during failing antiretroviral therapy Journal of Virology 2005 79 3 1772 1788 10.1128/JVI.79.3.1772-1788.2005 15650202 PMC544082

[B11] Smit TK Brew BJ Tourtellotte W Morgello S Gelman BB Saksena NK Independent evolution of human immunodeficiency virus (HIV) drug resistance mutations in diverse areas of the brain in HIV-infected patients, with and without dementia, on antiretroviral treatment Journal of Virology 2004 78 18 10133 10148 10.1128/JVI.78.18.10133-10148.2004 15331746 PMC515019

[B12] Wong JK Ignacio CC Torriani F Havlir D Fitch NJ Richman DD In vivo compartmentalization of human immunodeficiency virus: evidence from the examination of pol sequences from autopsy tissues J Virol 1997 71 3 2059 2071 9032338 10.1128/jvi.71.3.2059-2071.1997 PMC191294

[B13] Berman NG Parker RA Meta-analysis: neither quick nor easy BMC Med Res Methodol 2002 2 10 10 12171604 10.1186/1471-2288-2-10 PMC122061

[B14] Garvey L Winston A Walsh J Post F Porter K Gazzard B Fisher M Leen C Pillay D Hill T Antiretroviral therapy CNS penetration and HIV-1-associated CNS disease Neurology 2011 76 8 693 700 10.1212/WNL.0b013e31820d8b0b 21339496 PMC3053326

[B15] Finney DJ A statistician looks at met-analysis J Clin Epidemiol 1995 48 1 87 103; discussion 105-108 10.1016/0895-4356(94)00096-9 7853052

[B16] Hoenig JM Heisey DM The Abuse of Power: The Pervasive Fallacy of Power Calculations for Data Analysis The American Statistician 2001 55 1 1 6 10.1198/000313001300339860

[B17] Faul F Erdfelder E Buchner A Lang A-G Statistical power analyses using G*Power 3.1: Tests for correlation and regression analyses Behavior Research Methods 2009 41 4 1149 1160 10.3758/BRM.41.4.1149 19897823

[B18] Cohen J Statistical power analysis for the behavioral sciences 1988 2 New York: Academic Press

[B19] Cochran WG Sampling Techniques 1977 Wiley Inc

[B20] Cysique L Maruff P Brew B The neuropsychological profile of symptomatic, AIDS and ADC patients in the pre-HAART era: a meta-analysis Journal of the International Neuropsychological Society 2006 12 1 15 16903129 10.1017/s1355617706060401

[B21] Cysique LA Brew BJ Neuropsychological functioning and antiretroviral treatment in HIV/AIDS: a review Neuropsychol Rev 2009 19 2 169 185 10.1007/s11065-009-9092-3 19424802

[B22] May S Letendre S Haubrich R McCutchan JA Heaton R Capparelli E Ellis R Meeting practical challenges of a trial involving a multitude of treatment regimens: an example of a multi-center randomized controlled clinical trial in neuroAIDS J Neuroimmune Pharmacol 2007 2 1 97 104 10.1007/s11481-006-9057-8 18040832

[B23] Avison MJ Nath A Greene-Avison R Schmitt FA Greenberg RN Berger JR Neuroimaging correlates of HIV-associated BBB compromise J Neuroimmunol 2004 157 1-2 140 146 10.1016/j.jneuroim.2004.08.025 15579291

[B24] Wright EJ Grund B Robertson K Brew BJ Roediger M Bain MP Drummond F Vjecha MJ Hoy J Miller C Cardiovascular risk factors associated with lower baseline cognitive performance in HIV-positive persons Neurology 2010 75 10 864 873 10.1212/WNL.0b013e3181f11bd8 20702792 PMC2938971

[B25] Antinori A Giancola ML Grisetti S Soldani F Alba L Liuzzi G Factors influencing virological response to antiretroviral drugs in cerebrospinal fluid of advanced HIV-1-infected patients AIDS 2002 16 1867 1876 10.1097/00002030-200209270-00003 12351946

[B26] Chang L Ernst T Witt MD Ames N Walot I Jovicich J DeSilva M Trivedi N Speck O Miller EN Persistent brain abnormalities in antiretroviral-naive HIV patients 3 months after HAART Antivir Ther 2003 8 1 17 26 12713060

[B27] Cysique L Maruff P Brew B Antiretroviral therapy in HIV infection: are neurologically active drugs important? Archives of Neurology 2004 61 11 1699 1704 10.1001/archneur.61.11.1699 15534181

[B28] Cysique LA Maruff P Brew BJ Variable benefit in neuropsychological function in HIV-infected HAART-treated patients Neurology 2006 66 9 1447 1450 10.1212/01.wnl.0000210477.63851.d3 16682686

[B29] Cysique LA Vaida F Letendre S Gibson S Cherner M Woods SP McCutchan JA Heaton RK Ellis RJ Dynamics of cognitive change in impaired HIV-positive patients initiating antiretroviral therapy Neurology 2009 73 5 342 348 10.1212/WNL.0b013e3181ab2b3b 19474412 PMC2725933

[B30] De Luca A Ciancio BC Larussa D Murri R Cingolani A Rizzo MG Giancola ML Ammassari A Ortona L Correlates of independent HIV-1 replication in the CNS and of its control by antiretrovirals Neurology 2002 59 342 347 12177366 10.1212/wnl.59.3.342

[B31] Eggers C Hertogs K Sturenburg HJ van Lunzen J Stellbrink HJ Delayed central nervous system virus suppression during highly active antiretroviral therapy is associated with HIV encephalopathy, but not with viral drug resistance or poor central nervous system drug penetration Aids 2003 17 13 1897 1906 10.1097/00002030-200309050-00008 12960822

[B32] Marra CM Lockhart D Zunt JR Perrin M Coombs RW Collier AC Changes in CSF and plasma HIV-1 RNA and cognition after starting potent antiretroviral therapy Neurology 2003 60 1388 1390 12707454 10.1212/01.wnl.0000058768.73358.1a PMC2683839

[B33] Marra CM Zhao Y Clifford DB Letendre S Evans S Henry K Ellis RJ Rodriguez B Coombs RW Schifitto G Impact of combination antiretroviral therapy on cerebrospinal fluid HIV RNA and neurocognitive performance Aids 2009 23 11 1359 1366 10.1097/QAD.0b013e32832c4152 19424052 PMC2706549

[B34] Letendre S McCutchan J Childers M Woods S Lazzaretto D Heaton R Grant I Ellis R Group. H Enhancing antiretroviral therapy for human immunodeficiency virus cognitive disorders Annals of Neurology 2004 56 3 416 423 10.1002/ana.20198 15349869

[B35] Letendre S Marquie-Beck J Capparelli E Best B Clifford D Collier AC Gelman BB McArthur JC McCutchan JA Morgello S Validation of the CNS Penetration-Effectiveness rank for quantifying antiretroviral penetration into the central nervous system Arch Neurol 2008 65 1 65 70 10.1001/archneurol.2007.31 18195140 PMC2763187

[B36] Patel K Ming X Williams PL Robertson KR Oleske JM Seage GR Impact of HAART and CNS-penetrating antiretroviral regimens on HIV encephalopathy among perinatally infected children and adolescents Aids 2009 23 14 1893 1901 10.1097/QAD.0b013e32832dc041 19644348 PMC2821205

[B37] Sacktor N Tarwater PM Skolasky RL McArthur J Selnes OA Becker J Cohen B Miller EN CSF antiretroviral drug penetrance and the treatment of HIV-associated psychomotor slowing Neurology 2001 57 542 544 11502933 10.1212/wnl.57.3.542

[B38] Sevigny JJ Albert SM McDermott MP McArthur JC Sacktor N Conant K Schifitto G Selnes OA Stern Y McClernon DR Evaluation of HIV RNA and markers of immune activation as predictors of HIV-associated dementia Neurology 2004 63 2084 2090 15596754 10.1212/01.wnl.0000145763.68284.15

[B39] Smurzynski M Wu K Letendre S Robertson K Bosch RJ Clifford DB Evans S Collier AC Taylor M Ellis R Effects of central nervous system antiretroviral penetration on cognitive functioning in the ALLRT cohort AIDS 2011 25 3 357 365 10.1097/QAD.0b013e32834171f8 21124201 PMC3022370

[B40] Tozzi V Balestra P Salvatori MF Vlassi C Liuzzi G Giancola ML Giulianelli M Narciso P Antinori A Changes in cognition during antiretroviral therapy: comparison of 2 different ranking systems to measure antiretroviral drug efficacy on HIV-associated neurocognitive disorders J Acquir Immune Defic Syndr 2009 52 1 56 63 10.1097/QAI.0b013e3181af83d6 19731418

