# Supplementary material for: Central nervous system antiretroviral efficacy in HIV infection: a qualitative and quantitative review and implications for future research
Source: BMC Neurol. 2011 Nov 22;11:148. doi: 10.1186/1471-2377-11-148 (PMC3252248; doi:10.1186/1471-2377-11-148)
Supplement: Additional file 1 — Excluded studies per our criteria of exclusion. detailed references of the excluded studies. [file 1471-2377-11-148-S1.PDF]

Additional file 1

Excluded studies per our criteria of exclusion:

1. Gisolf EH, van Praag RM, Jurriaans S, Portegies P, Goudsmit J, Danner SA, Lange JM, Prins JM: **Increasing cerebrospinal fluid chemokine concentrations despite undetectable cerebrospinal fluid HIV RNA in HIV-1-infected patients receiving antiretroviral therapy**. *J Acquir Immune Defic Syndr* 2000, **25**(5):426-433.

2. Lafeuillade A, Solas C, Halfon P, Chadapaud S, Hittinger G, Lacarelle B: **Differences in the detection of three HIV-1 protease inhibitors in non-blood compartments: clinical correlations**. *HIV Clin Trials* 2002, **3**(1):27-35.

3. Brew BJ, Halman M, Catalan J, Sacktor N, Price RW, Brown S, Atkinson H, Clifford DB, Simpson D, Torres G *et al*: **Factors in AIDS dementia complex trial design: results and lessons from the abacavir trial**. *PLoS Clin Trials* 2007, **2**(3):e13.

4. Evers S, Rahmann A, Schwaag S, Frese A, Reichelt D, Husstedt IW: **Prevention of AIDS dementia by HAART does not depend on cerebrospinal fluid drug penetrance**. *AIDS Res Hum Retroviruses* 2004, **20**(5):483-491.

5. Dougherty RH, Skolasky R, McAthur JC: **Progression of HIV-associated Dementia treated with HAART**. *AIDS Read* 2002, **12**(2):69-74.

6. Garvey L, Winston A, Walsh J, Post F, Porter K, Gazzard B, Fisher M, Leen C, Pillay D, Hill T *et al*: **Antiretroviral therapy CNS penetration and HIV-1-associated CNS disease**. *Neurology* 2011, **76**(8):693-700.

7. Lanoy E, Guiguet M, Bentata M, Rouveix E, Dhiver C, Poizot-Martin I, Costagliola D, Gasnault J: **Survival after neuroAIDS: association with antiretroviral CNS Penetration-Effectiveness score**. *Neurology* 2011, **76**(7):644-651.
