# Supplementary material for: Central nervous system antiretroviral efficacy in HIV infection: a qualitative and quantitative review and implications for future research
Source: BMC Neurol. 2011 Nov 22;11:148. doi: 10.1186/1471-2377-11-148 (PMC3252248; doi:10.1186/1471-2377-11-148)
Supplement: Additional file 2 — Quality scoring table and instructions for NeuroHAART studies. This table and instructions are an exact reproduction of those designed for the blind review of studies. [file 1471-2377-11-148-S2.PDF]

Additional file 2

Quality scoring table and instructions for NeuroHAART studies

**Blind review for study # :**			
		**Circle**	**Comments**
**Study Design**	1. Is the study design described?	Yes No	
	2. Is the study design appropriate for the study questions?	Yes No	
	3. Are there clear inclusion/exclusion criteria?	Yes No	
	4. Is (are) the NeuroHAART score(s) clearly defined?	Yes No	
	5. Are the study time points (cross-sectional or longitudinal) clearly described?	Yes No	
**Study outcomes 1**	6. Are the outcomes clearly defined, including methods of measurements?	Yes No	
	7. Do the outcome measures answer the study questions?	Yes No	
**Study subjects**	8. Did the subjects meet the inclusion/exclusion criteria?	Yes No	
	9. Are demographics for all subject groups included?	Yes No	
	10. Are clinical characteristics for all subject groups included?		
	11. Are the study subjects homogeneous from a clinical standpoint?	Yes No	
**Controls**	12. If there are controls, or a comparison treatment group, are these comparable for demographic and clinical characteristics to the study subjects?	Yes No	
**Unblinded review # :**			
**Study outcomes 2**	13. If population parameters (e.g., norms) have been used where these appropriate?	Yes No	
	14. If the study was neurocognitive and longitudinal, did correction for practice effect was included?	Yes No	
	15. Are the study measurements validated and sensitive to test HIV-associated neurocognitive disorders?	Yes No	
	**Total Yes**		
	**Total NO**		
	Total applicable items		
	**% Quality score**		

Instructions for blind review:

For each study question from Q1 to Q12; review the study provided with a number and mark the number on the top of the page. Circle “Yes” or “No” for each study question and add any relevant comments that may help to clarify you answer as needed. Note that you need to read the extra instructions below to complete Q4; Q6; Q.10 and Q.11. If a question is not applicable then mark N/A in comments. The percentage score only retains the applicable questions.

Q4: The “Neurohaart score” is the score/definition representing the Central Nervous System penetrance or effectiveness of a group of antiretrovirals. The definition in the current paper that is inspected should be precise enough so that it could be easily replicated.

Q6. Note that the study outcomes can be neurocognitive/neuropsychological performance or CSF HIV RNA or both (see attached table).

Q10. The clinical characteristics should be looked for only in the HIV+ groups. As a guideline, value for the HIV disease stages are usually presented (CDC stage; CD4 cell count; nadir CD4 cell count and baseline rate of neuropsychological impairment when appropriate).

Q11. Does the study included HIV+ individuals with multiple neurological diagnostic conditions or only HIV+ individuals with and without HIV-associated neurocognitive disorder? (HIV-associated neurocognitive disorder encompasses all degree of severity from mild impairment to dementia).

Q13-Q15. These study points were examined by a neuropsychologist (L.C.) as follow: each study were inspected for whether neuropsychological cross-sectional norms or appropriate sample’s comparisons comparable for age, education and gender, as well as ethnicity when minority population have been included. CSF studies do not need normative data per se. Each longitudinal neurocognitive study was also inspected for correction of practice effect on neuropsychological performance. Each study outcomes measures were examined for validation and sensitivity to HAND. Size of the battery was also considered and small battery (battery composed with less than less than 8 tests were given a “NO” response on Q.15.
