# Supplementary material for: Central nervous system antiretroviral efficacy in HIV infection: a qualitative and quantitative review and implications for future research
Source: BMC Neurol. 2011 Nov 22;11:148. doi: 10.1186/1471-2377-11-148 (PMC3252248; doi:10.1186/1471-2377-11-148)
Supplement: Additional file 3 — Quality scores distribution in the 16 observational NeuroHAART studies. Figures and details of the quality scores obtained by each study. [file 1471-2377-11-148-S3.PDF]

## Additional file 3

## Quality scores distribution in the 16 observational NeuroHAART studies

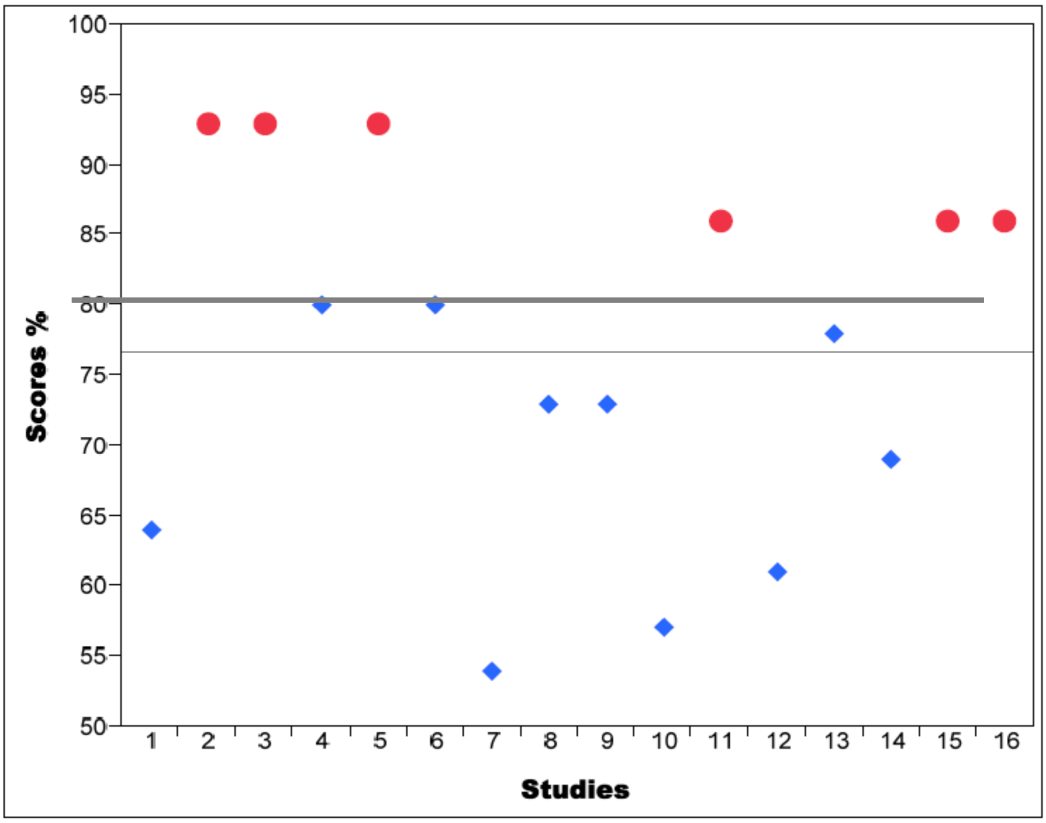


A score less than or equal to 80% (grey line cut-off) meant that a study presented at least three or more significant methodological limitations. This continuous score was developed to be more transparent than assessing quality categories such as "poor", "medium" or "high" quality.
