# Supplementary material for: Central nervous system antiretroviral efficacy in HIV infection: a qualitative and quantitative review and implications for future research
Source: BMC Neurol. 2011 Nov 22;11:148. doi: 10.1186/1471-2377-11-148 (PMC3252248; doi:10.1186/1471-2377-11-148)
Supplement: Additional file 4 — Details of the effect sizes' computations. Figure providing the effects sizes in the 6 studies with a quality score > 80%. [file 1471-2377-11-148-S4.PDF]

Additional file 4

Effect sizes computations

For all studies the effect sizes were derived using the Effect Sizes Determination Program [1]. This program allows estimation of effect sizes when only *p*-values when sample sizes are reported. When mean and standard deviation (SD) information were reported, we preferentially estimated the effect sizes using these data as recommended by [1].

Effect sizes were computed separately for cross-sectional and longitudinal studies with a quality score > 80%. No grand mean effect sizes were computed because the methodological approaches were too varied. Indeed, ideally pooled effect estimates should only be compiled using directly analogous studies [2-3]. Therefore, the effect sizes were only used to obtain a range of published effect sizes to then compute the best probable estimate of sample sizes for future studies. The effect sizes also provide a more appropriate metric than the *p*-value to assess the positive, neutral or negative impact of NeuroHAART [4].

Figure: Effects sizes in the 6 studies with a quality score > 80%

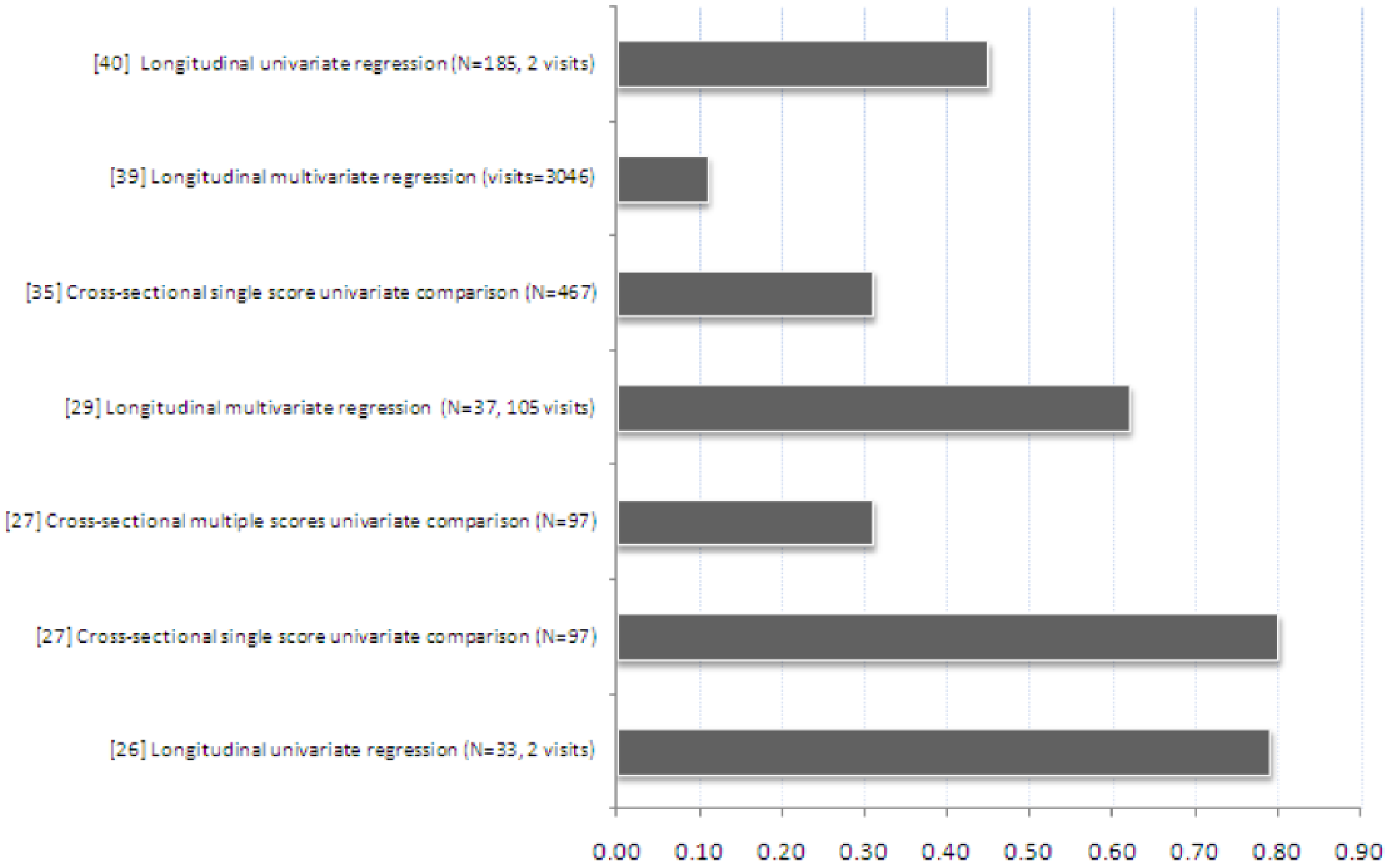

When a study reported significant and non-significant results for several NP tests, a mean effect sizes was computed for the significant results and a mean effect sizes for the non-significant results (i.e., [3].

If a study reported individual cognitive domain or individual NP tests scores as well as global scores, the global scores were retained for effect sizes' determination.

For [27] the effect size was computed for the neuropsychological data only.

For [29] and [39]: p-value reported on the CPE effect was derived from multivariate model adjusted for several other factors.

References

1. Lipsey M, Wilson D: **Practical meta-analysis**. London: Sage Publications; 2001.

2. Finney DJ: **A statistician looks at met-analysis**. *J Clin Epidemiol* 1995, **48**(1):87-103; discussion 105-108.

3. Stroup DF, Berlin JA, Morton SC, Olkin I, Williamson GD, Rennie D, Moher D, Becker BJ, Sipe TA, Thacker SB: **Meta-analysis of observational studies in epidemiology: a proposal for reporting. Meta-analysis Of Observational Studies in Epidemiology (MOOSE) group**. *JAMA* 2000, **283**(15):2008-2012.

4. Wilson DB: **Meta-analyses in alcohol and other drug abuse treatment research**. *Addiction* 2000, **95 Suppl 3**(3):S419-438.

5. Altman DG, Bland JM: **Absence of evidence is not evidence of absence**. *Bmj* 1995, **311**(7003):485.
